# Parathyroid Hormone‐Related Protein Inhibition Blocks Triple‐Negative Breast Cancer Expansion in Bone Through Epithelial to Mesenchymal Transition Reversal

**DOI:** 10.1002/jbm4.10587

**Published:** 2022-04-14

**Authors:** Jiarong Li, Anne Camirand, Mahvash Zakikhani, Karine Sellin, Yubo Guo, XiaoRui Luan, Catalin Mihalcioiu, Richard Kremer

**Affiliations:** ^1^ Centre for Translational Biology McGill University Health Centre Montréal QC Canada; ^2^ Third Affiliated Hospital Beijing University of Chinese Medicine Beijing China; ^3^ Department of Genetics, School of Medicine Zhejiang University Hangzhou China

**Keywords:** CANCER STEM CELL, EPITHELIAL TO MESENCHYMAL TRANSITION, PARATHYROID HORMONE‐RELATED PROTEIN, SKELETAL METASTASES, TRIPLE‐NEGATIVE BREAST CANCER

## Abstract

Parathyroid hormone‐related protein (PTHrP) plays a major role in skeletal metastasis but its action mechanism has not been fully defined. We previously demonstrated the crucial importance of PTHrP in promoting mammary tumor initiation, growth, and metastasis in a mouse model with a mammary epithelium‐targeted *Pthlh* gene ablation. We demonstrate here a novel mechanism for bone invasion involving PTHrP induction of epithelial to mesenchymal transition (EMT) and cancer stem cells (CSCs) regulation. Clustered regularly interspaced short palindromic repeats (CRISPR)‐mediated *Pthlh* gene ablation was used to study EMT markers, phenotype, and invasiveness in two triple‐negative breast cancer (TNBC) cell types (established MDA‐MB‐231 and patient‐derived PT‐TNBC cells). In vitro, *Pthlh* ablation in TNBC cells reduced EMT markers, mammosphere‐forming ability, and CD44^high^/CD24^low^ cells ratio. In vivo, cells were injected intratibially into athymic nude mice, and therapeutic treatment with our anti‐PTHrP blocking antibody was started 2 weeks after skeletal tumors were established. In vivo, compared to control, lytic bone lesion from *Pthlh* ‐ablated cells decreased significantly over 2 weeks by 27% for MDA‐MB‐231 and by 75% for PT‐TNBC‐injected mice (*p* < 0.001). Micro‐CT (μCT) analyses also showed that antibody therapy reduced bone lytic volume loss by 52% and 48% for non‐ablated MDA‐MB‐231 and PT‐TNBC, respectively (*p* < 0.05). Antibody therapy reduced skeletal tumor burden by 45% and 87% for non‐ablated MDA‐MB‐231 and PT‐TNBC, respectively (*p* < 0.002) and caused a significant decrease of CSC/EMT markers ALDH1, vimentin, and Slug, and an increase in E‐cadherin in bone lesions. We conclude that PTHrP is a targetable EMT molecular driver and suggest that its pharmacological blockade can provide a potential therapeutic approach against established TNBC‐derived skeletal lesions. © 2021 The Authors. *JBMR Plus* published by Wiley Periodicals LLC on behalf of American Society for Bone and Mineral Research.

## Graphical abstract

PTHrP inhibition reverses EMT in TNBC: in vitro, *Pthlh* ablation inhibits vimentin, Slug, ALDH1, and CD49f, enhances E‐cadherin, and lowers CD44/CD24 ratio and mammosphere formation, whereas our anti‐PTHrP mAb inhibits KI67 and cell survival and motility. In vivo, the mAb inhibits vimentin, Slug, and ALDH1 in TNBC xenografts and increases E‐cadherin, leading to growth inhibition of established TNBC bone tumors.

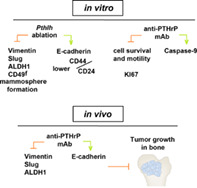

## Introduction

Triple‐negative breast cancers (TNBCs) generally strike younger patients and represent 12% to 24% of all diagnosed breast cancer cases. They are recognized as difficult to treat and carry poor prognosis and outcome.^(^ ^1^ ^)^ Although they are heterogeneous cancers, TNBCs share an absence of estrogen receptor, progesterone receptor, and human epidermal growth factor receptor 2 (HER‐2), and because of the lack of these receptors, there are currently no universal specific targets for their treatment.^(^ ^2^ ^)^ Tumor characteristics of TNBCs include rare histologies, high grade, elevated mitotic count, tumor necrosis, pushing margins of invasion, larger tumor size, and axillary node involvement.^(^ ^3^ ^)^ Despite chemotherapy, distant metastases often appear in lung, bone, liver, pleura, and brain, with the route of first metastasis correlating with patient survival.^(^ ^4^ ^)^ The prognosis is poor in TNBCs because of their frequent relapses and higher aggressiveness than receptor‐positive cancers.^(^ ^3^ ^)^ Indeed, less than 30% of women presenting with metastatic TNBC survive 5 years, and almost all of them die despite adjuvant chemotherapy.^(^ ^4^ ^)^ Although the exact mechanisms of TNBCs resistance have not been elucidated, they likely involve cancer stem cells (CSCs), because enrichment of cancer stem‐like cells with self‐renewing and tumor‐initiating capacities is associated with TNBC relapse.^(^ ^5^ ^)^

CSCs represent a small population of cancer cells able to seed new tumors, and they have been identified in most types of cancer including breast. The longevity, multilineage differentiation ability, drug resistance, and self‐renewal properties of CSCs are crucial characteristics when considering novel therapies. This can be explained in part because standard anti‐cancer treatments kill the majority of cancer cells and cause tumor debulking but fail to eliminate CSCs, which are left to survive, regenerate tumors locally, and disseminate to distal sites.^(^ ^6^, ^7^, ^8^ ^)^ Phenotypic characterization of human breast CSCs reveals expression of several markers of interest^(^ ^9^, ^10^ ^)^ with high tumor subtype variability^(^ ^11^ ^)^ and cell plasticity. CSCs may acquire or lose markers throughout tumor progression, compounding the difficulty of finding a universal breast CSC phenotype.^(^ ^12^ ^)^ Nevertheless, among the most characteristic markers for human breast CSCs are a high CD44^high^/CD24^low^ ratio and elevated aldehyde dehydrogenase‐1 (ALDH1) expression,^(^ ^13^ ^)^ presence of epithelial cell adhesion molecule/epithelium surface antigen (EpCAM/ESA), CD49^f^ (α6‐integrin), and the chemokine receptor CXCR4,^(^ ^8^, ^9^, ^14^, ^15^, ^16^ ^)^ which play roles in cellular adhesion, stem cell differentiation, and chemotherapy resistance.^(^ ^6^ ^)^ The acquisition of CSC characteristics is now recognized to be predominantly induced by a process called epithelial to mesenchymal transition (EMT).^(^ ^17^ ^)^

EMT is a cellular mechanism involved in normal physiological states and in pathological conditions such as wound healing, tissue fibrosis, and cancer.^(^ ^17^ ^)^ In tumor cells, EMT promotes the loss of epithelial properties and the acquisition of characteristics such as elongated phenotype, ability to degrade the extracellular matrix, loss of expression of adhesion protein E‐cadherin, and increased levels of the cytoskeletal protein vimentin that result in increased motility and invasion into the surrounding stroma.^(^ ^13^ ^)^ EMT activation increases stemness in carcinoma cells, allowing them to produce mammospheres in vitro and generate tumors when implanted in mouse hosts.^(^ ^17^, ^18^ ^)^ Importantly, the EMT process is also reversible to mesenchymal to epithelial transition (MET),^(^ ^18^ ^)^ allowing invading tumor cells that have reached the target tissue to return to an epithelial state and permitting cell–cell adhesion and micrometastasis formation.

Parathyroid hormone‐related protein (PTHrP) is a secreted factor expressed in almost all normal adult and fetal tissues. It is involved in a wide range of normal developmental and physiological processes but also plays a role in the progression of bone metastases,^(^ ^19^ ^)^ and its dysregulated expression in advanced cancers is the cause of malignancy‐associated hypercalcemia.^(^ ^20^, ^21^, ^22^ ^)^ Increased circulating levels of PTHrP in solid and hematological malignancies are associated with poor prognosis.^(^ ^23^, ^24^, ^25^, ^26^ ^)^ PTHrP is frequently overexpressed by breast and other solid tumors^(^ ^27^, ^28^, ^29^, ^30^, ^31^, ^32^, ^33^, ^34^ ^)^ and anti‐PTHrP antibodies have demonstrated efficacy against human breast cancer‐induced osteolysis in mice.^(^ ^35^, ^36^, ^37^ ^)^

Interestingly, nearly all human TNBC cell lines surveyed express PTHrP at high level, whereas most receptor‐positive lines do not (Appendix S1, Table S1). PTHrP displays complex and even paradoxical actions towards cancer depending on stage, tumor type, and microenvironment.^(^ ^38^ ^)^ For example, PTHrP expression in breast cancer patients has been linked to improved outcomes in a prospective patient cohort,^(^ ^39^ ^)^ and the ablation of PTHrP has been reported to increase tumor progression and inhibit animal survival in the MMTV‐neu mouse,^(^ ^40^ ^)^ a late‐onset mammary cancer model. However, most preclinical data support a pro‐tumorigenic role for PTHrP^(^ ^38^ ^)^; we recently examined tissue specimens from a cohort of treatment‐naive women newly diagnosed with TNBC and assessed that tumor cores from the majority of patients displayed very high PTHrP expression compared with normal breast samples. We found that PTHrP was a statistically significant independent prognostic factor for central nervous system progression‐free survival and for overall survival in TNBC patients.^(^ ^41^ ^)^ With therapeutic applications in view, our group has developed anti‐PTHrP blocking monoclonal antibodies (mAbs) efficient against human PTHrP. These blocking mAbs inhibit growth and metastasis of human PTHrP‐positive breast and prostate cancer^(^ ^42^, ^43^, ^44^ ^)^ and are strong inhibitors of TNBC cell growth in vitro, where they potentiate the effect of doxorubicin and taxol.^(^ ^45^ ^)^

Earlier studies highlighted the role of PTHrP as a major modulator of bone turnover in breast cancer metastasis.^(^ ^19^ ^)^ PTHrP released by bone‐invading tumor cells is now known to bind to its receptor on osteoblasts, activating the receptor activator of NF‐κB/RANK ligand (RANK/RANKL) system and releasing into the bone microenvironment several growth factors that feed cancer cells through a positive feedback loop.^(^ ^46^ ^)^ PTHrP promotes a favorable terrain allowing cancer cell expansion within bone, thereby supporting the “seed and soil” hypothesis proposed by Paget 130 years ago.^(^ ^47^ ^)^ More recently, we proposed a new paradigm based on our demonstration of PTHrP as a key regulator of tumor initiation and progression in the polyoma middle‐T oncoprotein mouse mammary tumor virus (PyMT‐MMTV), a CSC‐driven (early‐onset) cancer model^(^ ^48^, ^49^ ^)^ where we showed obligate involvement of PTHrP in the metastatic process.^(^ ^42^ ^)^ Moreover, a genomewide association analysis has identified a major locus for both estrogen receptor‐negative and receptor‐positive breast cancer susceptibility in a region (12p11) that contains the *Pthlh* gene.^(^ ^50^ ^)^ This combined evidence has led us to speculate that PTHrP may be an upstream control in events triggering development of breast EMT‐induced CSCs involved in tumor initiation and destined for metastasis. The identification of targetable TNBC molecular drivers is crucial to developing more efficient therapies. In the present study, we describe PTHrP control of the EMT process and of CSC levels, and we confirm the effectiveness of PTHrP blockade against established TNBC‐derived bone lesions.

## Materials and Methods

### Cell lines

MDA‐MB‐231 TNBC cells were obtained from ATCC (Manassas, VA, USA). The TNBC patient‐derived PTHrP‐positive line (PT‐TNBC) was isolated by our group following written patient consent approved by the Institutional Review Board of the McGill University Health Centre. PT‐TNBC tumor cells are negative for the estrogen receptor, progesterone receptor, and human epidermal growth factor receptor 2 (Appendix S1, Fig. S1). The PT‐TNBC primary tumor cells were preserved at early passages and transplanted into nude mice to maintain the culture. In vitro, cells were cultured at 37°C with 5% CO_2_ and 95% humidity in DMEM supplemented with 10% fetal bovine serum (FBS) for PT‐TNBC cells and in Roswell Park Memorial Institute (RPMI) medium supplemented with 10% FBS for MDA‐MB‐231 (Gibco, Grand Island, NY, USA).

### Growth assays

Cells were plated in 96‐well plates (1500–3000 cells per well) in triplicate and incubated in the medium described above in the previous section. After 24 hours, the complete medium was replaced with test medium (2% FBS) containing vehicle control or various doses of our anti‐PTHrP monoclonal antibody. After 72 hours at 37°C, PrestoBlue (Thermo Fisher, Montréal, QC, Canada; Cat # A13261) was added, plates were incubated at 37°C for 1 hour, and fluorescence readings were obtained on a TECAN Infinite 200 Pro microplate reader (Molecular Devices, Sunnyvale, CA, USA) at 560 to 570 nm excitation filter and 590 nm emission filter, according to the supplier's instructions.

### Animals

Athymic BALB/C nu/nu female mice were purchased from Charles River (Senneville, QC, Canada). All animal handling experiments were carried out in compliance with regulations of the McGill University Institutional Animal Care Committee and all surgeries were conducted in accordance with principles and procedures dictated by the highest standards of humane animal care.

### Construction of knockout TNBC cell lines by Clustered Regularly Interspaced Short Palindromic Repeats (CRISPR/Cas9)

**Fig 1:**
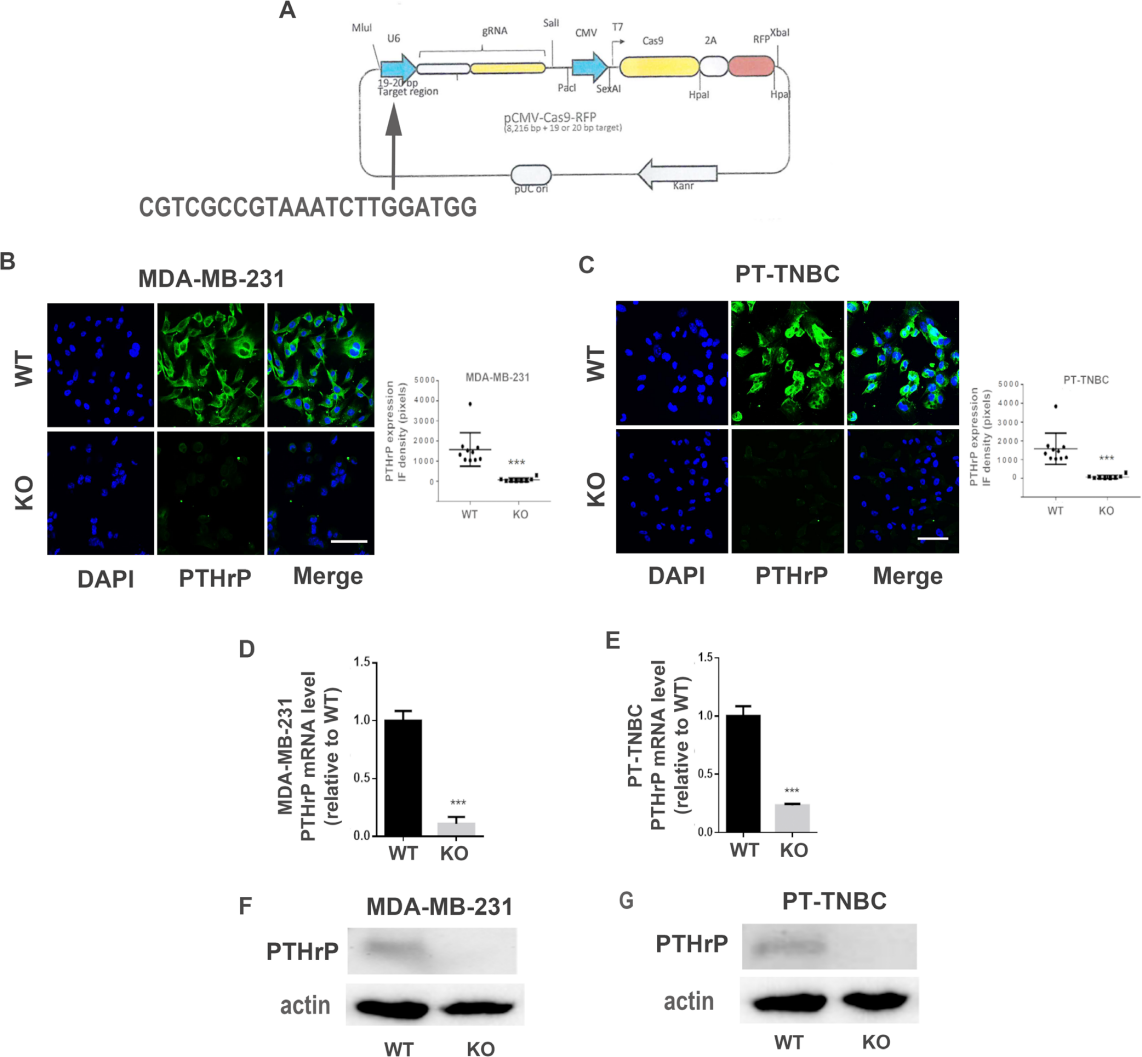
CRISPR‐mediated knockout of *Pthlh* gene in TNBC cell lines. (*A*) Diagram of the vector pCMVcas‐9‐RFP with human *Pthlh*‐specific RNA guide sequence. Blue arrows: promoters. The PTHrP exon 4‐specific 22‐nucleotide target sequence was inserted in the vector (vertical arrow at left). (*B*,*C*) IF staining for PTHrP in KO or control cell lines (empty vector) and fluorescence density diagrams (*n* = 10, *p* < 0.0001). (*D*,*E*) PTHrP mRNA levels in control or KO cell lines. (*F*,*G*) Western blot for PTHrP (17 kDa) and actin (43 kDa) in cell extracts from control or KO cell lines. Scale bars = 100 mm. 2A = 2A peptide link; Cas9 = caspase 9; gRNA = guide RNA; Kanr = kanamycin resistance; pUC ori = plasmid origin of replication; RFP = red fluorescent protein.

A U6G RNA‐Cas9‐2A‐red fluorescence protein vector (Invitrogen, Montréal, QC, Canada) was constructed with a human *Pthlh*‐specific guide RNA directed to exon 4 for knockout/KO), or an empty vector for control (*Pthlh* ^*WT*^ cells used as controls were transfected with the empty vector). The single guide RNA (sgRNA) sequence CGTCGCCGTAAATCTTGGATGG was inserted into the vector pCMVcas‐9‐RFP (Fig. 1*A*). The plasmid sequence was confirmed by sequencing at Genome Québec, McGill University (Montréal, QC, Canada). MDA‐MB‐231 and PT‐TNBC cells grown to 40% to 80% confluency in antibiotic‐free DMEM medium with 10% FBS were transfected with 2–3 μg of DNA using the PLUS transfection reagent (Thermo Fisher). Cells were grown 24 hours, trypsinized, and selected by fluorescence‐activated cell sorting (FACS) (FACS‐Aria II cell sorter; BD Biosciences, Mississauga, ON, Canada) for red fluorescent protein. Single clones were isolated using an autoMACS Pro Separator (Miltenyi Biotec, Somerville, MA, USA) and expanded in single wells (96‐well plate). DNA sequencing was done on isolated clones to confirm knockout status using the following primers:

*Pthlh*‐ exon 4 forward: CAAAAGAGCTGTGTCTGA

*Pthlh* – exon 4 reverse: TGAATCGAGCTCCAGCGACGTTGT

*Pthlh* exon3/4 forward: CGGTGTTCCTGCTGAGCTA

*Pthlh* exon 3/4 reverse: TGCGATCAGATGGTGAAGGA

Knockout (KO) at the proteomic level was confirmed by Western blot. Several *Pthlh* ^KO^ lines for MDA‐MD‐231 and PT‐TNBC displaying high PTHrP ablation were obtained.

### qRT‐PCR

RNA extractions were performed on cultured cells using the RNAeasy kit (Qiagen, Mississauga, ON, Canada) with on‐column DNAse treatment. First‐strand cDNA synthesis was performed on 500 ng total RNA with the RT^2^ First Strand kit (Qiagen). The cDNA template was combined with the RT^2^ Real‐Time SYBR Green/Rox master mix and RNAse‐free water (Roche, Mississauga, ON, Canada). Amplification was conducted on a 7300 Real‐Time PCR (Applied Biosystems, Foster, CA, USA) according to manufacturer's instructions. Housekeeping genes as well as reverse transcription and positive controls were included. Samples were normalized for relative quantification of expression by the comparative threshold cycle (2^−ΔΔ^CT) method using actin as an internal control.

List of primers (human):

PTHrP:forward: 5′‐GCCTCAA AAGAGCTGTGTCT‐3′

reverse: 5′‐GTTTCCTGAGTTAGGTATCTG‐3′

E‐cadherin:forward: 5′‐GGCGCCACCTCGAGAGA‐3′

reverse: 5′‐TGTCGACCGGTGCAATCTT‐3′

Slug:forward: 5′‐AAAAGCCAAACTACAGCGAACTG‐3′

reverse: 5′‐AGAATCTCTGCTTGTGGTATGACA‐3′

GAPDH:forward: 5′‐CATCCATGACAACTTTGGTATCGT‐3′

reverse: 5′‐CAGTCTTCTGGGTGGCAGTGA‐3′

ALDH1:forward: 5′‐TTTGTCCAGCCCACAGTGTT‐3′

reverse: 5′‐ACGCCATAGCAATTCACCCA‐3′

Vimentin:forward: 5′‐ACGTCTTGACCTTGAACGCA‐3′

reverse: 5′‐TCTTGGCAGCCACACTTTCA‐3′

### Western blotting

Western blotting was performed on cytosolic cellular extracts. Equal amounts of protein (50 μg) were fractionated by SDS‐PAGE electrophoresis, transferred to polyvinylidene fluoride (PVDF) membranes, and immunoblotted with rabbit anti human parathyroid hormone like hormone (PTHLH) polyclonal antibody (Ab) (Lifespan Biosciences, Seattle, WA, USA; Cat LS‐C408680) antibody. Image J software (NIH, Bethesda, MD, USA; https://imagej.nih.gov/ij/) was used for densitometric analysis.

### Anti‐PTHrP mAb

A PTHrP‐specific mAb against human PTHrP (1–33) peptide was generated^(^ ^42^ ^)^; hybridoma clone 158 produced a subclass immunoglobulin G3 (IgG3) mAb with strong binding to human PTHrP (1–33). The PA158 mAb is highly‐specific (no reaction with PTH), and has no cross‐reactivity with other fragments of the PTHrP protein. Clone158 has been deposited at the International Depositary Authority of Canada (accession number 060808‐02) and the US Patent Office (https://www.lens.org/lens/patent/US_7897139_B2). It has been validated in several publications.^(^ ^32^, ^42^, ^43^, ^44^, ^45^ ^)^

### Immunofluorescence and immunohistochemistry

Cultured cells were resuspended in PBS (1 × 10^6^ cells/mL), and 0.1‐mL aliquots deposited on SuperFrost Plus microscope slides (Thermo Fisher) using Shandon microfunnels (Thermo Fisher). The slides were air‐dried overnight, fixed with cold acetone for 10 minutes, air‐dried, stained with primary antibodies, and then reacted with fluorochrome‐conjugated secondary antibodies and 4′,6‐diamidino‐2‐phenylindole (DAPI). For breast tissues, slides were prepared automatically with a Ventana Discovery Ultra slide preparation instrument (Roche). Slides were deparaffinized, rehydrated, and antigen retrieval was performed with Ultra Cell Conditioning Solution (Ventana, Roche) for 30 minutes. Slides were incubated with primary antibodies at 37°C for 25 minutes. After washing, the secondary Ab was added and reacted 20 minutes at room temperature (RT). The slides were washed and ultraView Universal DAB detection kit (Ventana, Roche) was used to reveal the signal.

Primary antibodies used were as follows: PTHLH/PTHrP rabbit anti human Polyclonal Ab (Novus Biologicals, Littleton, CO, USA; NBP1‐59322), anti‐vimentin Ab (Cell Signaling Technology, Danvers, MA, USA; D21H3 XP 5741), anti‐human Slug polyclonal Ab (Invitrogen; PA5‐20289), anti‐ALDH1 polyclonal Ab (Invitrogen; PA5‐34901), anti‐E‐cadherin G‐10 monoclonal Ab (Santa Cruz Biotechnology, Santa Cruz, CA, USA; sc‐8426), anti‐CD44 human/mouse Ab (eBioscience, Montréal, QC, Canada; 17‐0441‐82), anti‐human CD24 10 (eBioscience; 11‐0247‐73), goat anti‐human anti‐Ki67 (Santa Cruz Biotechnology; sc‐23900), and rabbit anti‐caspase 9 (Novus Biologicals; NB100‐56118).

Secondary antibodies were all from Invitrogen: goat anti‐Mouse IgG (H+L) Cross‐Adsorbed Secondary Antibody, Alexa Fluor 568 (A‐11004), donkey anti‐Mouse IgG (H+L) Highly Cross‐Adsorbed Secondary Antibody, Alexa Fluor 488 (A‐21202), goat anti‐Rabbit IgG (H+L) Cross‐Adsorbed Secondary Antibody, Alexa Fluor 488 (A‐11008), donkey anti‐Rabbit IgG (H+L) Highly Cross‐Adsorbed Secondary Antibody, Alexa Fluor 546 (A10040), donkey anti‐Mouse IgG (H+L) Highly Cross‐Adsorbed Secondary Antibody, Alexa Fluor 647 (A‐31571), goat anti‐rabbit IgG (H+L) Highly Cross‐Adsorbed Secondary Antibody, Alexa Fluor 405 (A‐31556). Immunofluorescence (IF) results were analyzed with an LSM 780 Meta confocal microscope (Carl Zeiss Microimaging, Dorval, QC, Canada).

### Flow cytometry

Cells were harvested, washed, and numbers adjusted to 1 × 10^5^ to 5 × 10^5^ cells/mL in ice cold FACS buffer. 100 μL of cell suspension was stained in polystyrene round‐bottom 12 × 75 mm BD Falcon tubes with 0.1–10 μg/mL of the primary antibodies and incubated 30 minutes at 4°C in the dark. After washing, cells were analyzed using a LSRF Fortessa flow cytometer (BD Biosciences, Mississauga, ON, Canada). Antibodies used were: PE‐Cy 7 Mouse Anti‐Human CD24 (BD Pharmingen, Mississauga, ON, Canada; Cat #561646), Alexa Fluor 700 Mouse Anti‐Human CD44 (BD Pharmingen; Cat 561289), and CD49f (Integrin alpha 6) FITC Monoclonal Antibody (eBioscience; Cat #eBioGoH3).

### Mammosphere formation assay

MDA‐MB‐231 *Pthlh* ^KO^ or *Pthlh* ^WT^ cells from single clones were plated (4 × 10^3^ cells/well) into 48‐well ultra‐low attachment plates (Corning) in MammoCult medium (StemCell Technologies, Vancouver, BC, Canada) then incubated at 37°C for 7 days with three passages. Mammospheres were counted under phase‐contrast and the number of spheres larger than 60 μm in size was recorded. For IF, mammospheres were fixed in paraformaldehyde 4% for 15–30 minutes at RT, washed with PBS and permeabilized with 0.3% Triton X‐100 for 1 hour. Slides were incubated with the primary antibodies overnight, then washed and reacted with secondary Ab. Abs used were rabbit anti‐human vimentin monoclonal antibody SP20 (Invitrogen; Cat #CMA5‐14564), and EpCam CD326 monoclonal antibody 1B7 (Invitrogen; Cat #14‐9326‐82). Results were analyzed with an LSM 780 Meta confocal microscope (Carl Zeiss Microimaging).

### Wound healing (two‐dimensional migration assay)

A mechanical scratch was created with a pipette tip across confluent monolayer cultures of MDA‐MD‐231 and PT‐TNBC cells, and wound healing was quantified by measuring the shortest distance between edges at 0, 24, and 48 hours in three different fields per scratch.

### Intratibial injections

Human MDA‐MD‐231 or PT‐TNBC cells (*Pthlh* ^WT^ or *Pthlh* ^KO^) were injected (1 × 10^4^ in 15 μL of PBS) intratibially into 4‐week‐old to 5‐week‐old BALB/C nu/nu female mice (*n* = 10 mice/group). Briefly, the proximal end of the left tibia was exposed surgically and the knee was maintained in a flexed position. While grasping the ankle/leg of the mouse, a 25G needle was inserted into the tibia as a guide and a syringe with a 30G needle containing the cells was inserted inside the 25G. Starting 14 days after tumor cell injection, the animals received 200 μg anti‐PTHrP mAb intraperitoneally three times a week until euthanasia. Animals injected with MDA‐MB‐231 were euthanized at day 35 and mice with PT‐TNBC cells at day 28 due to tumor size. Control animals received injections of mouse IgG_3_ isotype (R&D Systems, Oakville, ON, Canada).

### X‐rays and micro‐computed tomography

X‐rays were taken at the McGill University Health Centre. The animals were placed in a supine position in an imaging tray and X‐rays of the hind limbs were taken with a Bruker In‐Vivo Xtreme optical imager. Scans were analyzed with ImageJ software. For micro–computed tomography (μCT), tibias were fixed in 10% formalin, scanned at the Center for Bone and Periodontal Research (McGill University), at ×40 magnification with a computer‐assisted bone histomorphometric analyzing system SkyScan 1072 (Bruker, Kontich, Belgium). Bone volume/total volume (BV/TV) analysis was performed on a region of interest (ROI) starting from the end of the growth plate and extending 300 cross‐sections toward the distal tibia (12.8 μm/slice, total distance 3.84 mm). Osteolytic volume was analyzed with bone analysis software (v. 2.2f; Skyscan, Aartselarr, Belgium).^(^ ^51^ ^)^ Contralateral bones (no TNBC cell injection) were used as controls (*n* = 7 mice per group).

### Goldner and tartrate‐resistant acid phosphatase stains

Three‐micrometer (3‐μm) sections of bone embedded in methylmethacrylate (J‐T Baker, Phillipsburg, NJ, USA) were placed on gelatin‐coated glass slides and stained with Goldner stain using standard protocols.^(^ ^51^ ^)^ Tumor areas within the bone cortex area were measured using Image J. For osteoclasts, detection of tartrate‐resistant acid phosphatase (TRAP) activity was carried out on 3‐μm bone sections according to the method of Liu and colleagues.^(^ ^52^ ^)^ Naphthol‐AS‐TR was used as substrate, and pararosaniline (Sigma‐Aldrich, Oakville, ON, Canada) as coupler.^(^ ^51^ ^)^ A subset of each group (*n* = 5–7) was selected for analysis. Slides were scanned using a Leica Aperio A1 Turbo digital pathology scanner (MUHC Research Institute Technology Platforms). Analyses were performed using Bioquant Osteo Image analysis software (Bioquant Image Analysis Corp., Nashville, TN, USA). The number of osteoclasts (N.Oc) within the region of interest was determined (N.Oc/bone surface mm^2^). Osteoblast surface (Ob,S/bone surface mm^2^) was also determined from these sections.

### Statistical analysis

Numerical data are presented as the mean ± standard error of the mean (SE). The data were analyzed by ANOVA followed by a Bonferroni's post‐test to determine the statistical significance of differences. All statistical analyses were performed using Instat Software (GraphPad Software), and *p* < 0.05 was considered statistically significant.

### Study approval

These animal studies were approved by McGill University's Animal Compliance Office, institutional approval number 7713.

## Results

### CRISPR‐mediated *Pthlh* gene ablation

The CRISPR/Cas9 technology was used to ablate exon 4 of the *Pthlh* gene from MDA‐MB‐231 and PT‐TNBC cells (Fig. 1*A*). The empty vector was used for control. After CRISPR ablation, PTHrP is almost undetectable by IF in *Pthlh* ^KO^ cells compared to *Pthlh* ^WT^ controls (IF density 1581 ± 264 for MDA‐MB‐231 *Pthlh* ^WT^ versus 74 ± 33 for *Pthlh* ^KO^, and 1257 ± 209 for PT‐TNBC *Pthlh* ^WT^ versus 74 ± 23 for *Pthlh* ^KO^; *n* = 9, *p* < 0.0001; Fig. 1*B*,*C*). This was confirmed by qPCR: *Pthlh* ^KO^ cells expressed 11.0 ± 0.016% and 22.8 ± 0.068% of *Pthlh* mRNA measured in *Pthlh* ^WT^ (for MDA‐MD‐231 and PT‐TNBC, respectively; *n* = 10, *p* < 0.0001) (Fig. 1*D*,*E*). PTHrP protein concentration in cell lysates estimated by Western blot indicate that *Pthlh* ^KO^ cells express only 13.6% to 15.0% of the PTHrP protein compared to *Pthlh* ^WT^ for MDA‐MB‐231 and PT‐TNBC, respectively (densitometry results: 1672 ± 300 pixels for *Pthlh* ^WT^ versus 228 ± 70 for *Pthlh* ^KO^ in MDA‐MB‐231s, and 1407 ± 70 pixels for *Pthlh* ^WT^ versus 211 ± 52 for *Pthlh* ^KO^ in PT‐TNBCs; *n* = 10, *p* < 0.001) (Fig. 1*F*,*G*, full Western blot in Appendix S1, Fig. S2). These results confirm efficient removal of PTHrP expression in both MDA‐MB‐231 and PT‐TNBC cells.

### *Pthlh* gene deletion reverts TNBC cells to epithelial morphology and phenotype

**Fig 2:**
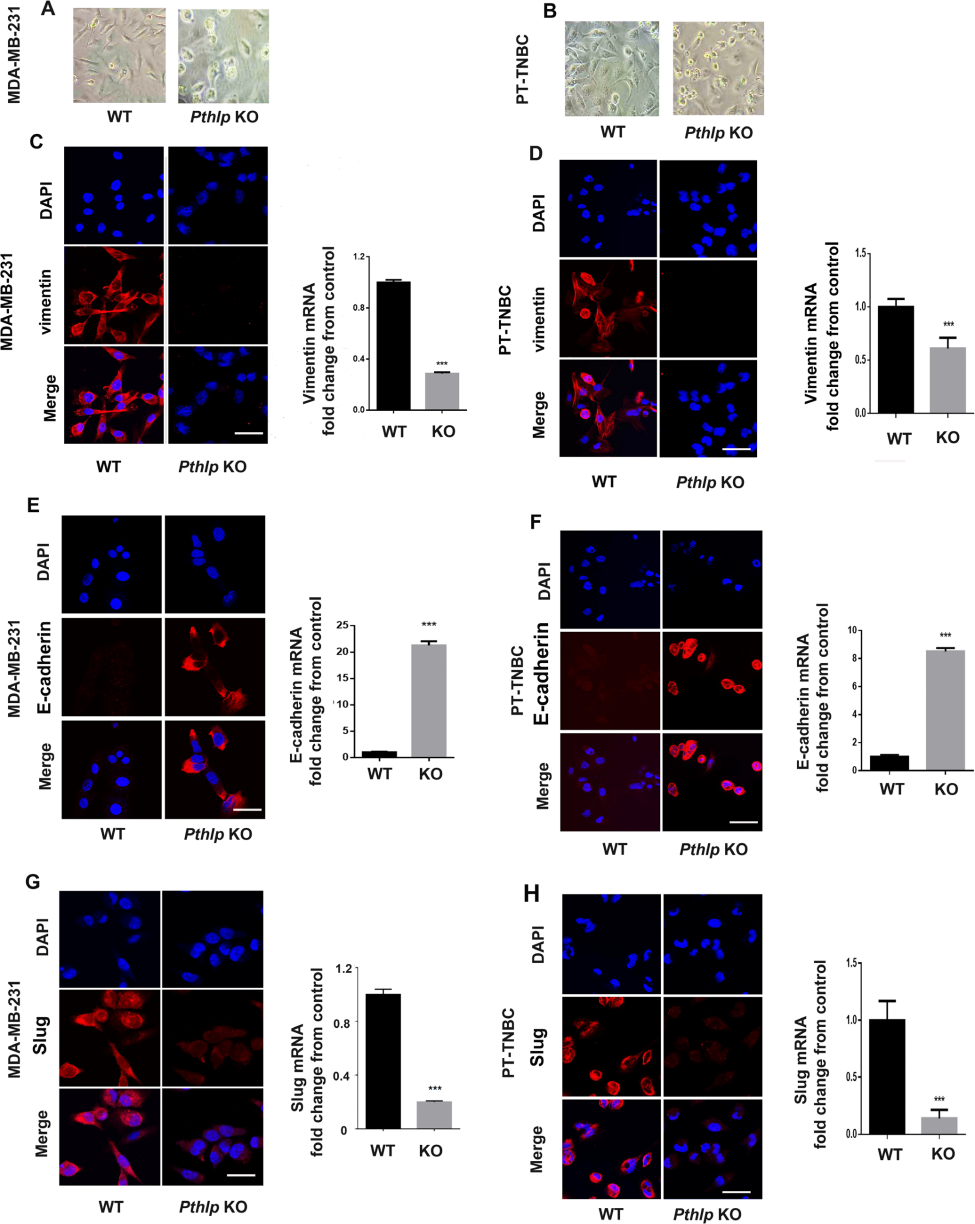
*Pthlh* gene ablation reverts TNBC cells to epithelial morphology and phenotype. (*A*,*B*) Phase contrast microscopy of *Pthlh* ^WT^ (empty vector) and *Pthlh* ^KO^ MDA‐MB‐231 (left) and PT‐TNBC cells (right). (*C*,*D*) IF detection and mRNA levels of vimentin. (*E*,*F*) IF detection and mRNA levels of E‐cadherin. (*G*,*H*) IF detection and mRNA levels of Slug in *Pthlh* ^WT^ and *Pthlh* ^KO^ cells. DAPI: blue; vimentin: E‐cadherin; Slug: red. Scale bars = 100 μm, *n* = 9, *p* < 0.001.

Ablation of the *Pthlh* gene in MDA‐MB‐231 and PT‐TNBC cells was accompanied by a change in cell morphology. Whereas *Pthlh* ^WT^ cells displayed the typical elongated mesenchymal phenotype of invasive cells, *Pthlh* ^KO^ cells shifted to a rounded morphology encountered in epithelial conditions (Fig. 2*A*,*B*). Furthermore, in MDA‐MB‐231 cells, E‐cadherin expression increased by more than 20‐fold (21.29‐fold ±0.25), whereas mesenchymal cell surface marker vimentin and transcription factor Slug (which downregulates E‐cadherin) decreased in *Pthlh* ^KO^ (to 28.67% ± 0.36% and 20.03% ± 0.24% of *Pthlh* ^WT^ levels, respectively). For PT‐TNBCs, E‐cadherin increased in *Pthlh* ^KO^ by 8.52‐fold ±0.23, vimentin decreased to 61.0% ± 0.075%, and Slug decreased to 22.1% ± 0.075% of *Pthlh* ^WT^ levels (*n* = 9, *p* < 0.001), Fig. 2*C*–*H*). These results suggest a crucial role for PTHrP in maintaining the mesenchymal state of the TNBC cells.

### *Pthlh* ablation reduces the CSC subpopulation in MBA‐MB‐231 and PT‐TNBC cells

**Fig 3:**
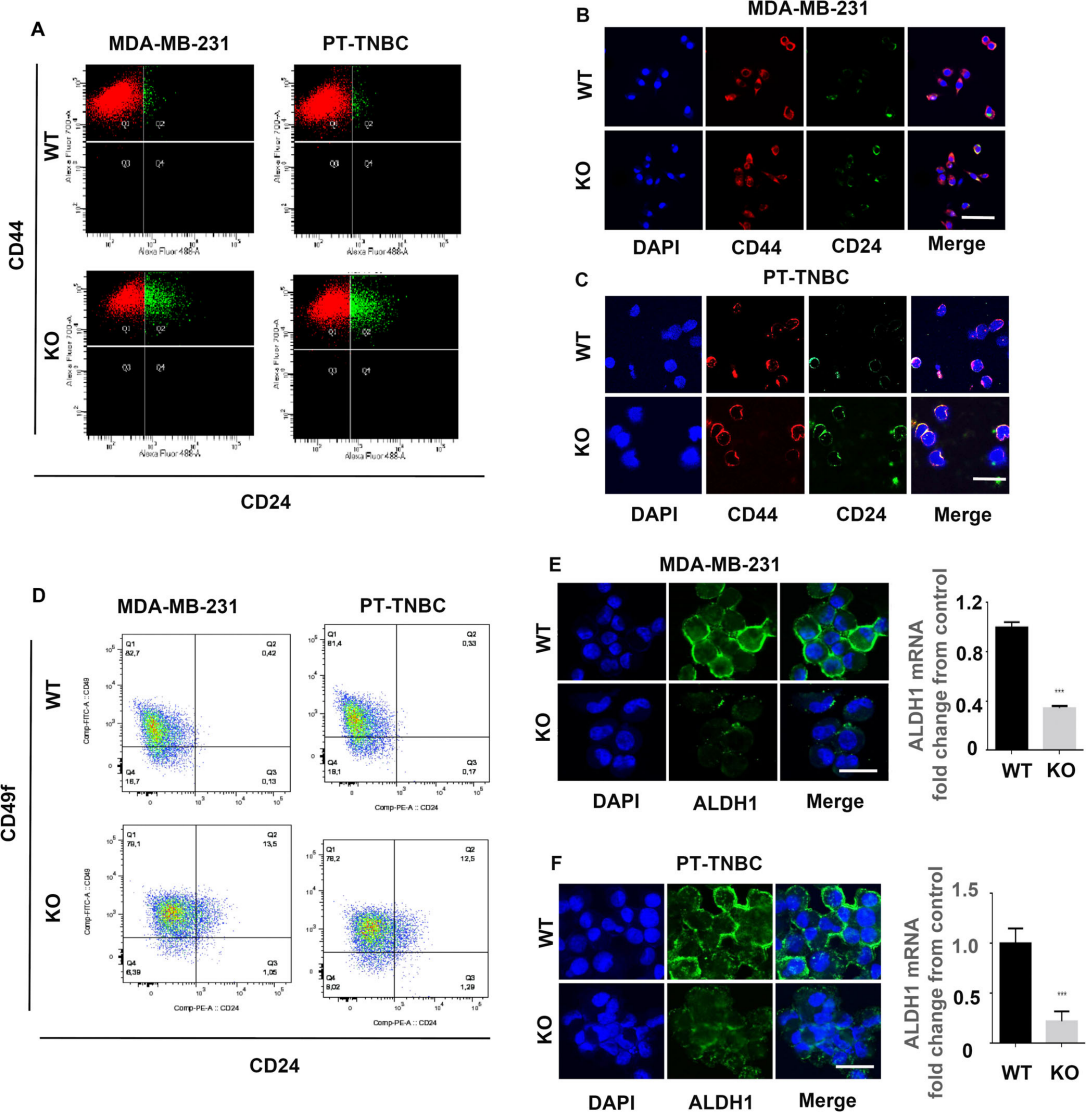
*Pthlh* ablation reduces the CSC subpopulation in MBA‐MB‐231 and PT‐TNBCs cells. (*A*) Flow analysis of CD44 and CD24‐expressing MDA‐MB‐231 (left) and PT‐TNBC cells (right). (*B*,*C*) IF staining for CD44 and CD24 in WT and KO MDA‐MB‐231 and PT‐TNBCs. (*D*) Flow analysis of CD49f and CD24 in MDA‐MB‐231 (left) and PT‐TNBC cells (right). (*E*,*F*) IF staining for ALDH1 and mRNA levels in WT (empty vector) and KO MDA‐MB‐231 and PT‐TNBCs. DAPI: blue; CD44: red; CD24: green; ALDH1: green. *n* = 5, *p* < 0.001. Scale bars = 100 μm (*B*,*C*), 50 μm (*E*,*F*).

Among the CSC markers associated with breast pathology in humans are a high CD44/CD24 ratio, and elevated ALDH1 and CD49^f^ markers.^(^ ^53^, ^54^, ^55^, ^56^ ^)^ The CD44^high^/CD24^low^ status is an indication of breast cancer cell malignancy and of proliferation and tumorigenesis capacity.^(^ ^53^ ^)^ A very significant increase in CD24^high^ cells (green) in *Pthlh* ^KO^ cells was detectable by flow cytometry (Fig. 3*A*). The ratio of CD24^high^ cells to total population increased from 1.2% ± 0.01% to 13.18% ± 2.35% between WT and KO cells in MDA‐MB‐231 (*n* = 5, *p* = 0.0304). For PT‐TNBC, the increase was from 1.3% ± 0.01% in WT to 12.88% ± 2.47% in KO (*n* = 5, *p* = 0.0377). The consequence of this increase in CD24^high^ cells is a decrease in the CD44^hi^/CD24^lo^ ratio in KO cells with respect to WT cells. IF staining confirmed the shift in both cell lines (Fig. 3*B*,*C*). In MDA‐MB‐231, *Pthlh* ablation caused an increase in the ratios of CD44^hi^ CD24^hi^/CD49^f hi^ cells from 0.374% ± 0.015% to 13.02% ± 0.193%, and a decrease in CD44^hi^ CD24^lo^/CD49^f lo^ cells from 17.00% ± 0.31% to 6.11% ± 0.32%. In PT‐TNBCs, *Pthlh* ablation caused an increase in the ratios of CD44^hi^ CD24^hi^/CD49^f hi^ cells from 0.35% ± 0.011% to 12.05% ± 0.21%, and a decrease in CD44^hi^ CD24^lo^/CD49^f lo^ cells from 18.10% ± 0.3142% to 8.02%± 0.27% (*n* = 5, *p* < 0.001, Fig. 3*D*). IF staining in *Pthlh* ^WT^ and *Pthlh* ^KO^ MDA‐MB‐231 and PT‐TNBC cells revealed a decrease in ALDH1 expression in *Pthlh* ^KO^ cells to 34.27% ± 0.59% of *Pthlh* ^WT^ in MDA‐MB‐231 with respect to *Pthlh* ^WT^cells and 22.0% ± 0.15% in PT‐TNBCs (*n* = 9, *p* < 0.001 Fig. 3*E*,*F*). These results point to a loss of cancer stem cell characteristics markers consequent to *Pthlh* ablation in TNBC cells.

### *Pthlh* ablation inhibits mammosphere formation capacity in MBA‐MB‐231 and PT‐TNBC cells

**Fig 4:**
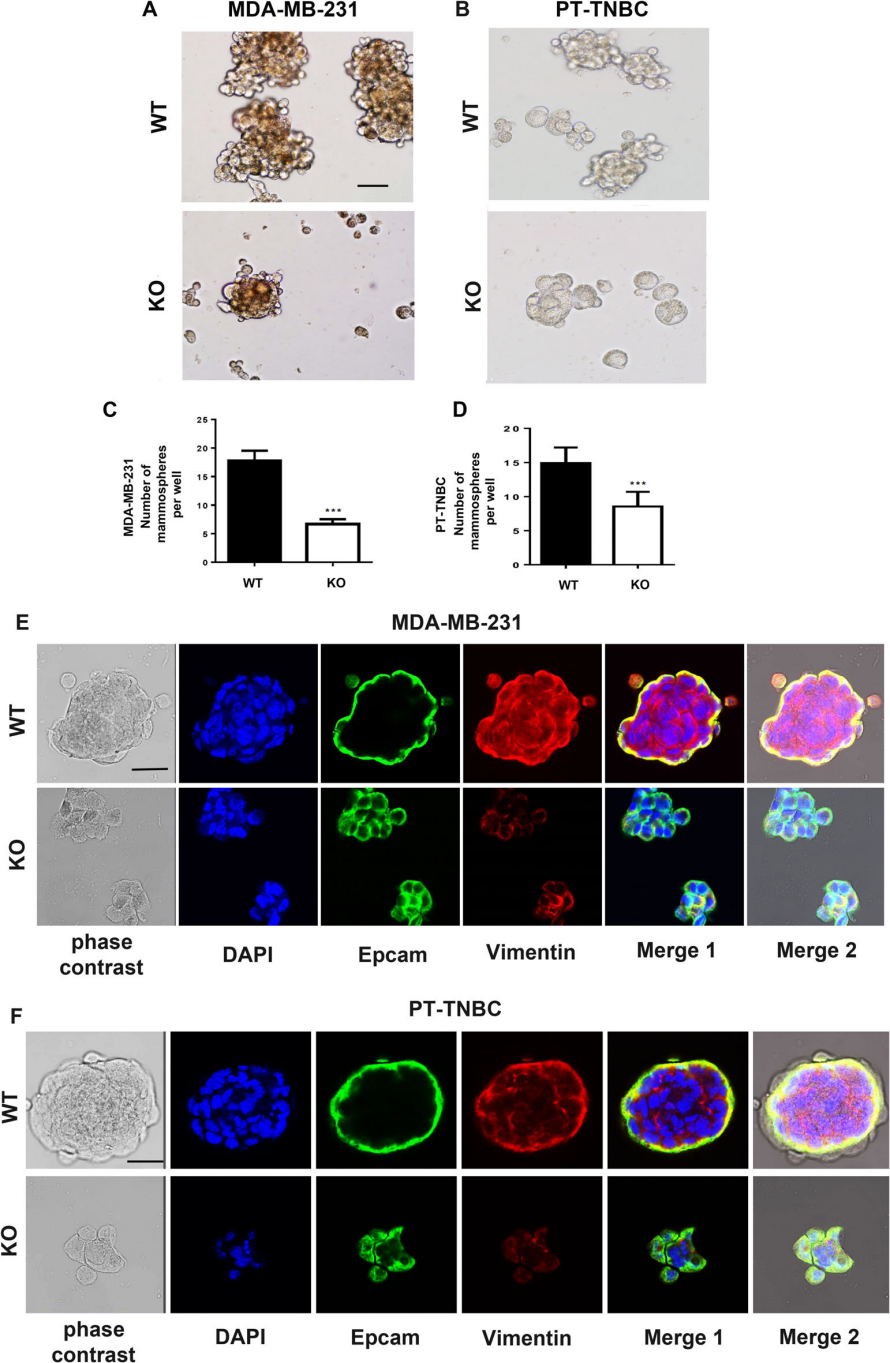
*Pthlh* ablation inhibits mammosphere formation capacity in MBA‐MB‐231 and PT‐TNBC cells. Bright field microscopy of primary mammospheres from MDA‐MB‐231 (*A*) and PT‐TNBC (*B*) *Pthlh* ^WT^ (empty vector, top) and *Pthlh* ^KO^ cells (below). Mammosphere counts per well for MDA‐MB‐231 (*C*) and PT‐TNBC (*D*). (*n* = 22, *p* < 0.0001). EpCAM and vimentin IF staining in mammospheres from *Pthlh* ^WT^ and *Pthlh* ^KO^ cells for MDA‐MB‐231 (*E*) and PT‐TNBC (*F*). DAPI: blue; EpCAM: green; vimentin: red. Scale bars = 100 μm.

Primary mammospheres develop in culture from single breast cancer stem cell clones and are a hallmark of invasive capacity. Here, *Pthlh* ablation decreased the capacity for mammosphere formation by 50% to 60% in TNBC cell lines with respect to *Pthlh* ^WT^ cells (*n* = 22, *p* < 0.0001) (Fig. 4*A*–*D*). IF staining of mammospheres revealed that cells maintained the epithelial cell adhesion marker (Epcam) as a marker of cancer stem cells, but lost expression of the mesenchymal cell surface marker vimentin when *Pthlh* was ablated (Fig. 4*E*,*F*). These in vitro observations indicate a diminished mammosphere formation capacity and therefore a decrease in invasiveness in TNBC cells consequent to *Pthlh* ablation.

### PTHrP blocking mAb reduces cell division and migration capacity and increases apoptosis of MBA‐MB‐231 and PT‐TNBC cells

**Fig 5:**
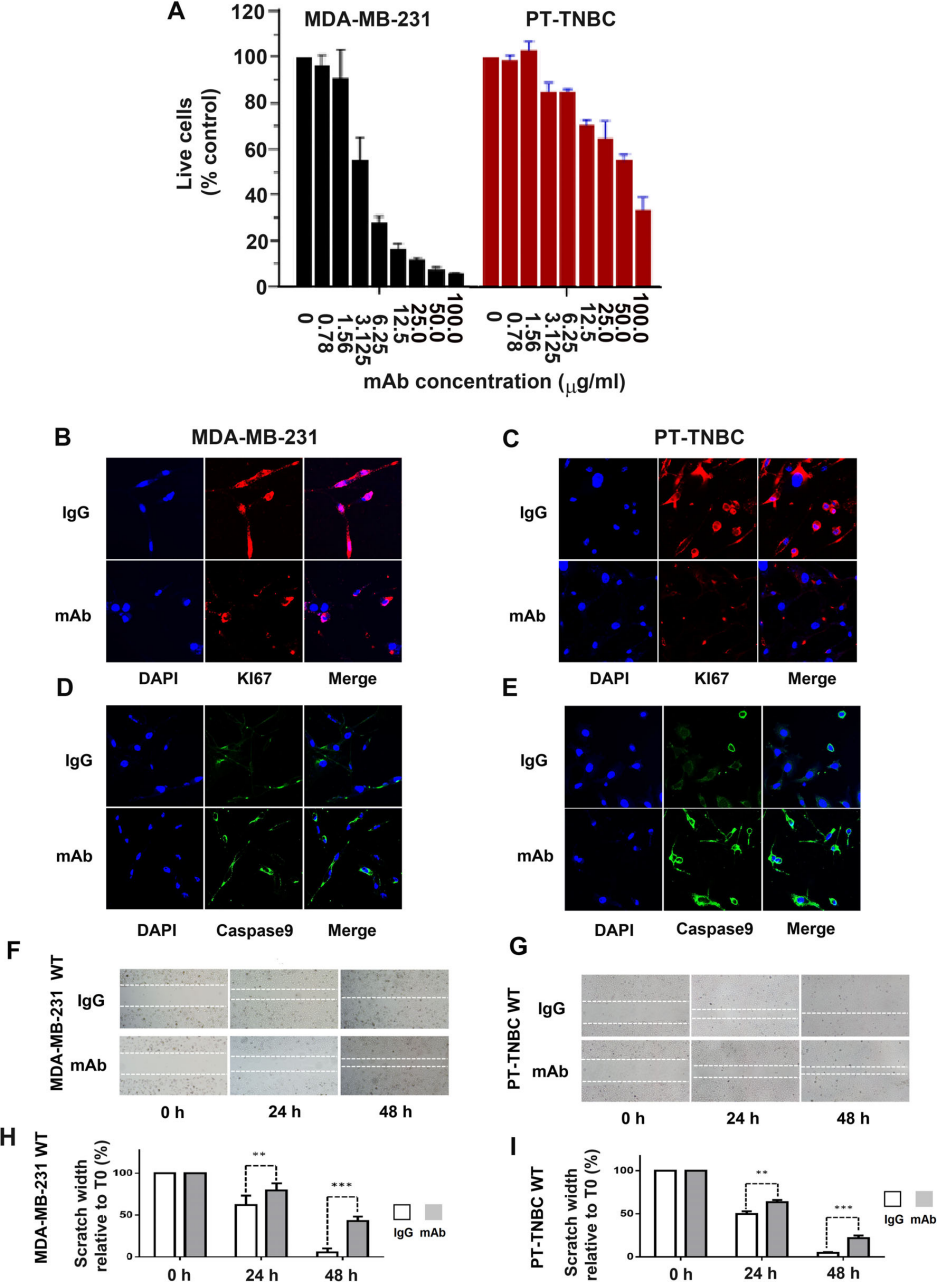
Anti‐PTHrP mAb reduces cell division and migration capacity and increases apoptosis in TNBC cells. (*A*) Reduction in percentage of live cells of MDA‐MB‐231 and PT‐TNBC *Pthlh* ^WT^ in vitro (72 hours) by increasing concentrations of anti‐PTHrP mAb. (*B*,*C*) 72‐Hour treatment with anti‐PTHrP mAb (2 μg/mL) reduces proliferation marker KI67 expression with respect to IgG controls for *Pthlh* ^WT^ MDA‐MB‐231 and PT‐TNBC cells. (*D*,*E*) 72‐Hour treatment with anti‐PTHrP mAb (2 μg/mL) increased apoptosis marker caspase 9 expression with respect to IgG controls for *Pthlh* ^WT^ MDA‐MB‐231 and PT‐TNBC cells. (*F*–*I*) Scratch test and width values for *Pthlh* ^WT^ MDA‐MB‐231 and PT‐TNBC cells treated with control IgG or anti‐PTHrP mAb. (*n* = 9, *p* < 0.001). DAPI: blue; caspase 9: green; KI67: red. Scale bars = 100 μm.

A reduction in percentage of live cells was observed in both *Pthlh* ^WT^ MDA‐MB‐231 and patient‐derived PT‐TNBC primary cells in vitro after 72 hours of treatment with our blocking anti‐PTHrP mAb (*n* = 3, *p* < 0.001, Fig. 5*A*). The mAb treatment (2 μg/mL) resulted in inhibition of proliferation marker Ki67 expression and stimulation of caspase 9 apoptosis marker (*n* = 3, *p* < 0.001, Fig. 5*B*–*E*). In a wound healing assay, these cell lines displayed a substantial reduction of migration capacity after treatment with the same anti‐PTHrP mAb (2 μg/mL). After 24 hours, scratch width (relative to time 0) in MDA‐MB‐231 cells was 62.27% ± 3.73% for IgG‐treated cells, and 79.38% ± 2.87% for anti‐PTHrP mAb‐treated cells. After 48 hours, scratch width (relative to time 0) in MDA‐MB‐231 cells was 5.7% ± 1.7% for IgG‐treated cells, and 43.07% ± 1.7% for anti‐PTHrP mAb‐treated cells (*n* = 9, *p* < 0.001, Fig. 5*F*,*H*). For PT‐TNBC cells, scratch width at 24 hours (relative to time 0) was 49.77% ± 3.15% for IgG‐treated cells, and 63.51% ± 2.58% for anti‐PTHrP mAb‐treated cells. After 48 hours, scratch width (relative to time 0) was 4.68% ± 0.86% for IgG‐treated cell (*n* = 9, *p* < 0.001, Fig. 5*G*,*I*). Because there are ~80% live cells left after 72 hours of exposure to 2 μg/mL mAb (Fig. 5*A*), the 24‐hour and 48‐hour scratch tests indicate migration speed is the main factor affected, although some apoptosis is involved, as suggested by caspase‐9 induction (Fig. 5*D*,*E*). These results indicate that treatment of *Pthlh* ^WT^ TNBC cells in monoculture with our anti‐PTHrP mAb significantly decreases cell division and migration capacity and induces apoptosis.

### In vivo treatment with anti‐PTHrP mAb counters established skeletal lesions from TNBC cell lines

Nude athymic female mice were injected intratibially with MDA‐MD‐231 or PT‐TNBC cells (either *Pthlh* ^WT^ empty vector, or *Pthlh* ^KO^). When bone tumors were established (14 days after cell injection), half the *Pthlh* ^WT^ mice started receiving intraperitoneally our anti‐PTHrP mAb and the other half a control IgG (three times per week, protocol diagram in Appendix S1, Fig S3*A*). Our mAb appears to have no negative side effects because animal well‐being as reflected by body weight was maintained throughout treatment (no significant difference, *p* > 0.05, Appendix S1, Fig S3*B*,*C*).

**Fig 6:**
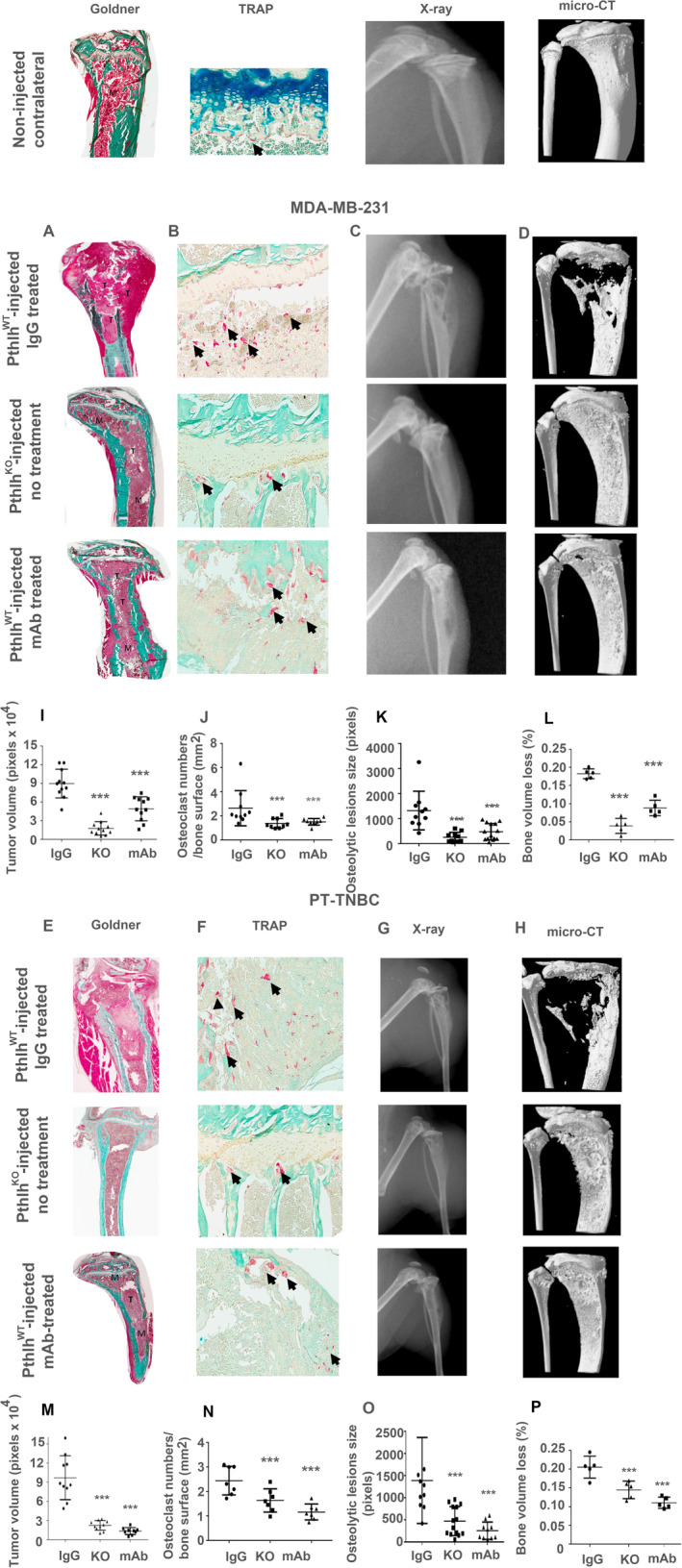
In vivo treatment with anti‐PTHrP mAb counters established skeletal lesions from TNBC cell lines. (Top) Contralateral bone (non‐injected). (*A*–*D*) MDA‐MB‐231 injected mice. (*E*–*H*) PT‐TNBC injected mice. (*A*,*E*): Goldner stain (*n* = 11, *p* < 0.0001), (*B*,*F*): TRAP stain for osteoclasts (arrows) (*n* = 5, *p* < 0.001), (*C*,*G*): X‐ray at euthanasia (*n* = 10, *p* < 0.001), (*D*,*G*): μCT (*n* = 5, *p* < 0.0001). (Top row): *Pthlh* ^WT^ xenografts with IgG control treatment, (middle row): *Pthlh* ^KO^ xenografts no treatment. (third row): *Pthlh* ^WT^ xenografts (empty vector) with blocking anti‐PTHrP mAb treatment. (*I*–*P*) Histograms of values at euthanasia. M = bone marrow; T = tumor.

At euthanasia, histomorphometry by Goldner staining and TRAP staining as well as X‐ray and μCT analyses were performed. The contralateral tibial bones (that received no TNBC cell injection) are shown at top left in Fig. 6. Non‐injected bone (no cancer cells) is not statistically significantly affected by anti‐PTHrP mAb treatment in terms of osteoblast numbers/mm^2^ (1.875 ± 0.211 for anti‐PTHrP mAb treatment versus 1.813 ± 0.181 for control IgG, *n* = 8, *p* = 0.989) or osteoclast numbers/mm^2^ (1.577 ± 0.233 for anti‐PTHrP mAb treatment versus 1.445 ± 0.077 for control IgG, *n* = 8, *p* = 0.474), or bone volume/total volume (BV/TV: 41.5% ± 1.3% for anti‐PTHrP mAb treatment versus 39.8% ± 1.2% for control IgG, *n* = 7 *p* = 0.338). (Appendix S1, Fig. S4).

As expected, when tibias were injected with *Pthlh* ^WT^ cells, severe bone lesions calculated by Goldner staining appeared in animals treated with the control IgG (Fig. 6*A* for MDA‐MD‐231 and Fig. 6*E* for PT‐TNBC). Treatment of the established lesions with our mAb caused a substantial reduction in tumor volume estimated by Goldner staining (by 45% and 87%, for MDA‐MB‐231 and PT‐TNBC, respectively). For MDA‐MB‐231 *Pthlh* ^WT^‐injected mice, tumor surface was calculated at 89700 ± 6940 pixels for IgG‐treated animals, and 49100 ± 5810 pixels for anti‐PTHrP mAb‐treated injected animals. For comparison, injection of *Pthlh* ^KO^ cells in mice showed tumor volume reduction by 87% (15700 ± 3200 pixels) (*n* = 11, *p* = 0.0002). For PT‐TNBC *Pthlh* ^WT^‐injected mice, tumor surface was calculated at 97061 ± 10873 pixels for IgG‐treated animals and 13450 ± 1815 pixels for anti‐PTHrP mAb‐treated animals (*n* = 10, *p* < 0.0001). For mice injected with *Pthlh* ^KO^ cells, tumor surface was 26200 ± 2100 pixels, a decrease of 74%.

TRAP staining (Fig. 6*B* for MDA‐MD‐231) reveals 2.634 ± 0.460 osteoclasts per bone surface (mm) in IgG‐treated mice versus 1.501 ± 0.091 in mAb‐treated *Pthlh* ^WT^‐injected animals and 1.376 ± 0.136 for *Pthlh* ^KO^‐injected mice (*n* = 6, *p* < 0.05). For PT‐TNBC *Pthlh* ^WT^‐injected mice (Fig. 6*F*), TRAP staining reveals 2.442 ± 0.219 osteoclasts per bone surface (mm) in IgG‐treated mice versus 1.160 ± 0.123 in mAb‐treated animals. For mice injected with *Pthlh* ^KO^ cells, osteoclasts numbers were 1.633 ± 0.179, a decrease of 54% (*n* = 7, *p* < 0.01).

Bone lytic activity was calculated by X‐ray at euthanasia (Fig. 6*C* for MDA‐MD‐231). At euthanasia, administration of our anti‐PTHrP mAb had decreased the rate of osteolytic progression in animals injected with MDA‐MB‐231 by 27%. (Lysis area 2659 ± 750 pixels for control IgG treatment versus 1946 ± 820 pixels for anti‐PTHrP mAb). Lytic activity in *Pthlh* ^KO^‐injected mice was reduced by 88% with 320 ± 120 pixels (*n* = 10, *p* < 0.05 and *p* < 0.001). In PT‐TNBC‐injected mice (Fig. 6*G*), the mAb decreased osteolytic progression by 75%. (Lysis area 1440 ± 808 pixels for control IgG treatment versus 366 ± 145 pixels for anti‐PTHrP mAb, *n* = 10, *p* < 0.001). For mice injected with *Pthlh* ^KO^ cells, ablation decreased osteolytic progression by 66% (480 ± 380 pixels).

μCT analysis (Fig. 6*D* for MDA‐MD‐231) indicates bone loss values of 0.1825 ± 0.006 in control IgG‐treated mice versus 0.0881 ± 0.009 in mAb‐treated MDA‐MB‐231‐injected animals, a decrease of 52% due to treatment (*n* = 5, *p* < 0.0001). In mice injected with *Pthlh* ^KO^ cells, bone loss was reduced by 78% (0.0410 ± 0.009). For PT‐TNBC‐injected mice (Fig. 6*H*), μCT analysis indicates values of 0.2054 ± 0.013 in control IgG‐treated mice versus 0.1104 ± 0.006 in mAb‐treated animals (*n* = 5, *p* < 0.001), indicating decreased bone volume loss activity of 48% for mice injected with PT‐TNBC cells. In mice injected with *Pthlh* ^KO^ cells, bone loss was reduced by 32% (0.141 ± 0.009). Figure 6*I*–*P* shows all results for MDA‐MB‐231 and PT‐TNBC.

These results show that anti‐PTHrP mAb in vivo treatment of established tumors in animals injected with *Pthlh* ^WT^ TNBC cells significantly reduces the rate of progression and the size of established bone lesions, and maintains better bone structure than control injections, in a manner similar to outcomes due to injection of *Pthlh* ^KO^ cells; however, the blocking mAb therapeutic intervention can be more efficient than *Pthlh* ablation. Importantly, our mAb appears to have no negative side effects on animal well‐being (Appendix S1, Fig. S3*B*,*C*) and no significant effect on non‐injected bone volume or osteoclast and osteoblast numbers in the experimental conditions described here.

### In vivo treatment with blocking anti‐PTHrP mAb causes EMT reversal in established TNBC‐derived bone lesions

**Fig 7:**
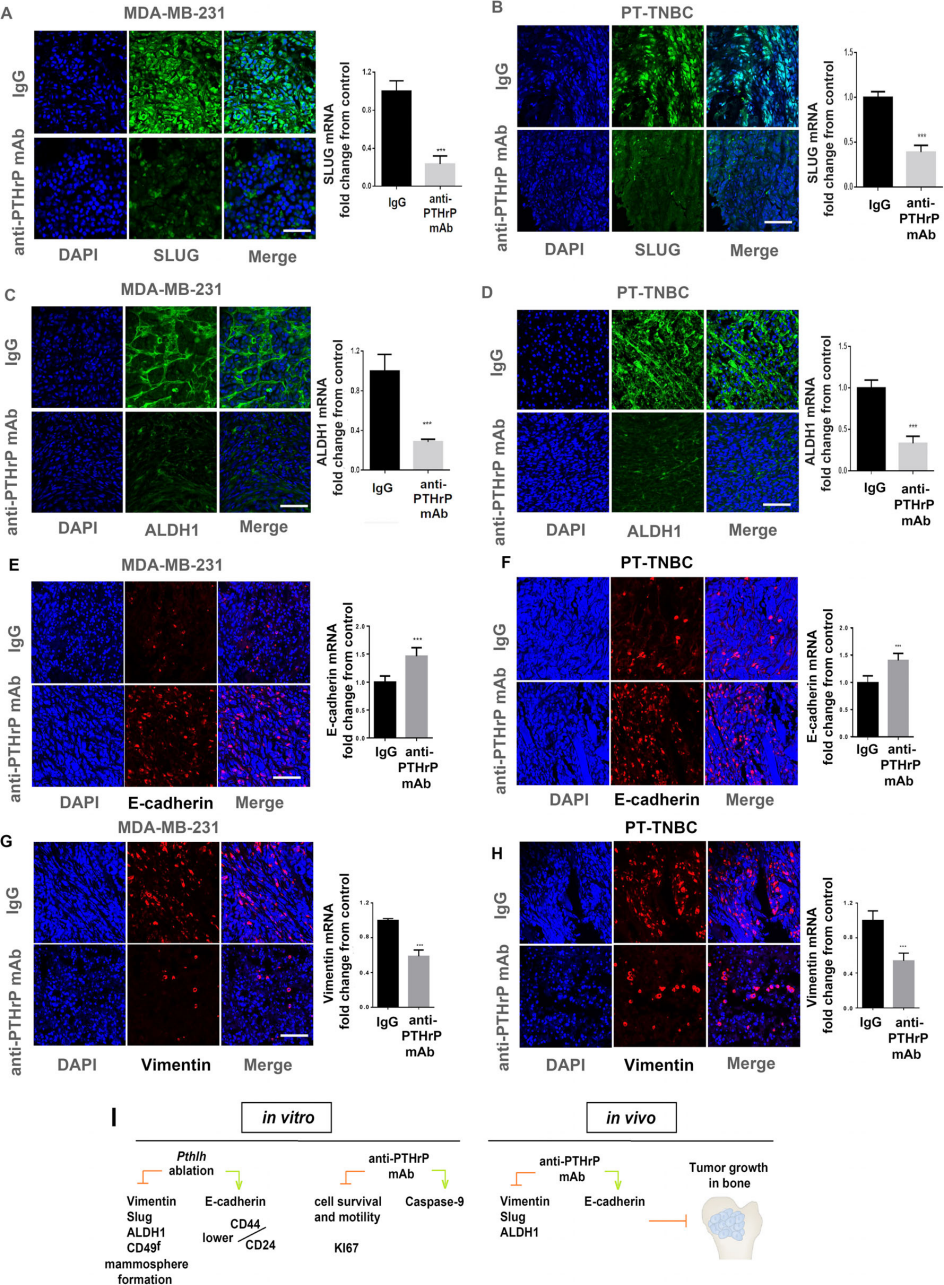
Inhibition of EMT and CSC markers in bone lesions following in vivo administration of anti‐PTHrP mAb: IF staining and mRNA level for Slug in MDA‐MB‐231 *Pthlh* ^WT^ injected mice (*A*) and PT‐TNBC *Pthlh* ^WT^ injected mice (*B*). IF staining and mRNA level for ALDH1 in MDA‐MB‐231 *Pthlh* ^WT^ injected mice (*C*) and PT‐TNBC *Pthlh* ^WT^ injected mice (*D*). IF staining and mRNA level for E‐cadherin in MDA‐MB‐231 *Pthlh* ^WT^ injected mice (*E*) and PT‐TNBC *Pthlh* ^WT^ injected mice (*F*). IF staining and mRNA level for vimentin in MDA‐MB‐231 *Pthlh* ^WT^ injected mice (*G*) and PT‐TNBC *Pthlh* ^WT^ injected mice (*H*). Scale bar = 100 mm, *n* = 10, *p* < 0.001. (*I*) Diagram summarizing mechanistic involvement of PTHrP in EMT and CSC number control and therapeutic implications. In TNBC cells in vitro, *Pthlh* ablation inhibits vimentin, Slug, ALDH1, and CD49^f^, enhances E‐cadherin and lowers CD44/CD24 ratio and mammosphere formation, while our anti‐PTHrP mAb inhibits KI67 and cell survival and motility. In vivo, the mAb inhibits vimentin, Slug, and ALDH1 in TNBC xenografts and increases E‐cadherin, leading to growth inhibition of established TNBC bone tumors.

Tumor cells extracted from bone marrow at euthanasia were stained for the mesenchymal transcription factor Slug (cadherin and vimentin regulator), E‐cadherin and vimentin, and for the CSC marker ALDH1. In the skeletal tumors of animals injected with MDA‐MB‐231 and treated with our anti‐PTHrP mAb, the expression of Slug, ALDH1, and vimentin were greatly reduced (to 23.2% ± 12%, 28.7% ± 6%, and 58.6% ± 3.6% for Slug, ALDH1, and vimentin, respectively, *n* = 10, *p* < 0.001). For bone tumors of mice injected with PT‐TNBCs, Slug, ALDH1, and vimentin expression was similarly reduced to 41.2% ± 14%, 33.0% ± 18%, and 54.0% ± 2.8%, respectively (*n* = 9, *p* < 0.001). The anti‐PTHrP mAb treatment increased E‐cadherin expression to 146.0% ± 3.0% in MDA‐MB‐231 and to 138.0% ± 5.1% for PT‐TNBC cells (*n* = 9, *p* < 0.001, Fig. 7*A*–*H*). These results suggest a decrease in EMT status within the bone marrow tumors following mAb treatment of established *Pthlh* ^WT^ TNBC lesions.

## Discussion

Metastatic breast cancer kills more than 40,000 individuals every year in the United States.^(^ ^57^ ^)^ Effective approaches are therefore urgently needed for countering breast cancer that has spread to distal sites. Bone is the most frequent metastatic site for breast cancer, and is invaded in 70% of metastatic patients.^(^ ^58^ ^)^ Although osteoclastic inhibitors such as bisphosphonates and the RANKL inhibitor denosumab are approved in the treatment of skeletal metastases to limit bone loss and reduce skeletal‐related events,^(^ ^19^ ^)^ these drugs only target the “soil” (bone microenvironment) aspect of the skeletal metastases and do not address the “seed” portion of the equation; ie, the invading tumor cells. As a result, these therapies are not curative and action against the invading cells is required to optimize therapeutic efficacy. PTHrP is detected in about 70% of breast cancer‐derived bone metastases, and is elevated in more than 60% of breast cancer primary tumors.^(^ ^59^ ^)^ This sets PTHrP as a “seed” factor that can be targeted in addition to the “soil” to potentially enhance therapeutic efficacy in patients.

In the present study, we report novel mechanisms involving EMT and CSCs that drive the deleterious effect of PTHrP expression in the “seed” and the efficacy of PTHrP blockade in established bone lesions (Fig. 7*I*). The malignant phenotype of TNBC cells has been reported to be suppressed by epigenetic reprogramming of EMT.^(^ ^60^ ^)^ Here, EMT reversal is accomplished in “seed” by CRISPR ablation of the *Pthlh* gene in TNBC cells. The absence of PTHrP expression is accompanied by a morphological reshaping characteristic of EMT reversal, concomitantly with a decrease in the expression of the Slug transcription factor and cytoskeletal protein vimentin, as well as an increase in E‐cadherin expression indicating a return to the epithelial state characterized by a loss of motility and migratory properties. This evidence is consistent with a role for PTHrP in EMT regulation, with *Pthlh* ablation reversing EMT to MET. PTHrP has previously been associated with EMT‐MET control in normal kidney tubuloepithelial cells,^(^ ^61^, ^62^ ^)^ normal intestinal epithelial cells,^(^ ^63^ ^)^ and in prostate cancer cells,^(^ ^64^ ^)^ but this had not yet been demonstrated in breast cancer. Transforming growth factor beta (TGF‐β) has a proven central role in breast cancer metastasis to the skeleton where the cytokine induces EMT, with host‐derived TGF‐β acting on tumor cells using PTHrP as an effector.^(^ ^65^ ^)^ We confirm here that PTHrP is likely a crucial intermediate in this process because its absence reverses EMT and significantly reduces the invasive motile state of TNBC cells.

*Pthlh* ablation in TNBC cells is also accompanied here by inhibition of expression of the CSC markers ALDH1 and CD49^f^, as well as by a decreased CD44/CD24 ratio consequent to significant elevation in the CD24 cell population. The CD44^high^/CD24^low^ subpopulation of breast cancer cells displays stem/progenitor cell properties and exhibits enhanced invasive properties. These cells are crucial to metastatic progression^(^ ^54^ ^)^ and conversely, elevation of CD24 with respect to CD44 indicates a reduction in tumorigenesis capacity.^(^ ^53^, ^54^ ^)^ Furthermore, the very significant decrease in mammosphere‐forming ability seen in *Pthlh* ^KO^ cells supports reduced invasive capacity. This set of observations dovetails well with reports of a PTHrP role in control of several metabolic pathways that power CSC growth: sonic hedgehog, Notch, Wnt, TGF‐β, and Bmi1.^(^ ^5^, ^8^, ^16^, ^66^, ^67^, ^68^, ^69^, ^70^, ^71^ ^)^ Downregulation of CSC‐associated genes Casp3 and Tert, as well as upregulation of Sox2, has been reported for MCF‐7 cells overexpressing PTHrP.^(^ ^72^ ^)^ However, our study is the first report of the first report of direct PTHrP regulation of CSC induction in vitro and in vivo.

A link between EMT and CSC was first established by Mani and colleagues,^(^ ^73^ ^)^ who demonstrated that exposure of normal mammary epithelial cells to EMT‐inducing transcription factors Snail or Twist caused the cells to adopt the CD44^high^/CD24^low^ expression profile, mesenchymal morphology, and surface markers, and display enhanced mammosphere‐forming capacity. Recent evidence reinforces the theory that CSCs are the main drivers of cancer progression and determinants of therapeutic response. Furthermore, the phenotypic differences that distinguish CSCs and the non‐CSCs that comprise the bulk of tumor cells now appear to be consequent to the induction of EMT in CSCs.^(^ ^17^ ^)^ The implications are important because this suggests that CSCs are tumor cells that acquire stem cell‐like properties as a consequence of a regulatable EMT^(^ ^74^ ^)^ and we show here that PTHrP plays a crucial role in this process.

In the experimental conditions used here, the in vivo intraperitoneal treatment of mice (tibially‐injected with *Pthlh* ^WT^ cells) with our anti‐PTHrP did not induce osteoporotic‐like features in terms of osteoblast and osteoclast numbers as well as bone volume loss in non‐injected bone as had been observed in homozygous or heterozygous *Pthlh*‐ablated mice.^(^ ^75^, ^76^ ^)^ The in vivo mAb treatment nevertheless displayed an inhibitory effect on established lesions through processes involving EMT reversal and decrease in the CSC marker ALDH1, similar to that observed in the primary cells through *Pthlh* ablation. From a therapeutic point of view, the fact that PTHrP is able to regulate EMT‐induced CSC phenotype in breast cancer suggests it is a valuable therapeutic target for preventing recurrence and metastasis, although further studies will be needed for confirmation.

PTHrP has been reported to display complex and sometimes opposite actions toward cancer and its actions likely depend on tumor type, stage, and metastatic microenvironment. Nevertheless, most preclinical data support a pro‐tumorigenic role for PTHrP,^(^ ^38^ ^)^ and the present study illustrates a mechanistic link between PTHrP expression and EMT‐driven CSC phenotype in TNBC tumor cells (seed) as well as bone marrow (soil). We demonstrate here that anti‐PTHrP mAb treatment reverses EMT and has efficacy against established TNBC‐derived skeletal lesions.

## Disclosures

RK is the inventor of the therapeutic monoclonal anti‐PTHrP antibodies used in this study and authorized for use by Biochrom Pharma. RK holds an equity stake in Biochrom Pharma. All other authors declare no potential conflicts of interest.

## Supporting information


**Appendix** S1. Supporting Information.
Fig. S1
Fig. S2
Fig. S3
Fig. S4
Table S1
