# Supplementary material for: Parathyroid Hormone‐Related Protein Inhibition Blocks Triple‐Negative Breast Cancer Expansion in Bone Through Epithelial to Mesenchymal Transition Reversal
Source: JBMR Plus. 2022 Apr 14;6(6):e10587. doi: 10.1002/jbm4.10587 (PMC9189913; doi:10.1002/jbm4.10587)

**Supplementary materials, Li *et al***

**Supplementary table 1** : Almost all breast cancer cell lines reported to highly-express PTHrP are TNBCs. M: mesenchymal-like.

From: Guise *et al* (1996) J. Clin. Invest. 98:1544-1549, Keklikoglou *et al* (2012) Oncogene 31: 4150–4163. Subtype information from: Lehmann *et al* (2011) J. Clin. Invest. 121(7):2750–2767, Neve et al (2006) Cancer Cell 10:515–527.

| PTHrP high or very high expression | | | | | PTHrP low or very low expression | | | | |
| --- | --- | --- | --- | --- | --- | --- | --- | --- | --- |
| Cell line | Type | Gene  cluster | TNBC  sub-type |  | | Cell line | Type | Gene cluster |  |
| BT-549 | TNBC | Basal B | M |  | | MCF-7 | ER+PR+ | Luminal |  |
| Hs578-T | TNBC | Basal B | M |  | | SKRBR-3 | Her2+ | Luminal |  |
| MCF-10A | TNBC | Basal B | - |  | | T47-D | ER+PR+ | Luminal |  |
| MDA-MB-231 | TNBC | Basal B | M |  | | ZR75-1 | ER+ | Luminal |  |
| MDA-MB-435 | TNBC | Basal B | - |  | | BT-483 | ER+PR+ | Luminal |  |

**Supplementary figure S1:** **Biopsies of the primary breast tumor from PT-TNBC patient:** The PT-TNBC cell line isolated in our lab is derived from a triple negative breast tumor. Top row: IHC positive controls from various positive patients. Bottom row: patient-derived PT-TNBC cells are from an ER-negative, PR-negative and Her2-negative tumor. Staining done with antibodies against HER2 (human epidermal growth factor receptor 2, left), ER (estrogen receptor, center) and PR (progesterone receptor, right).


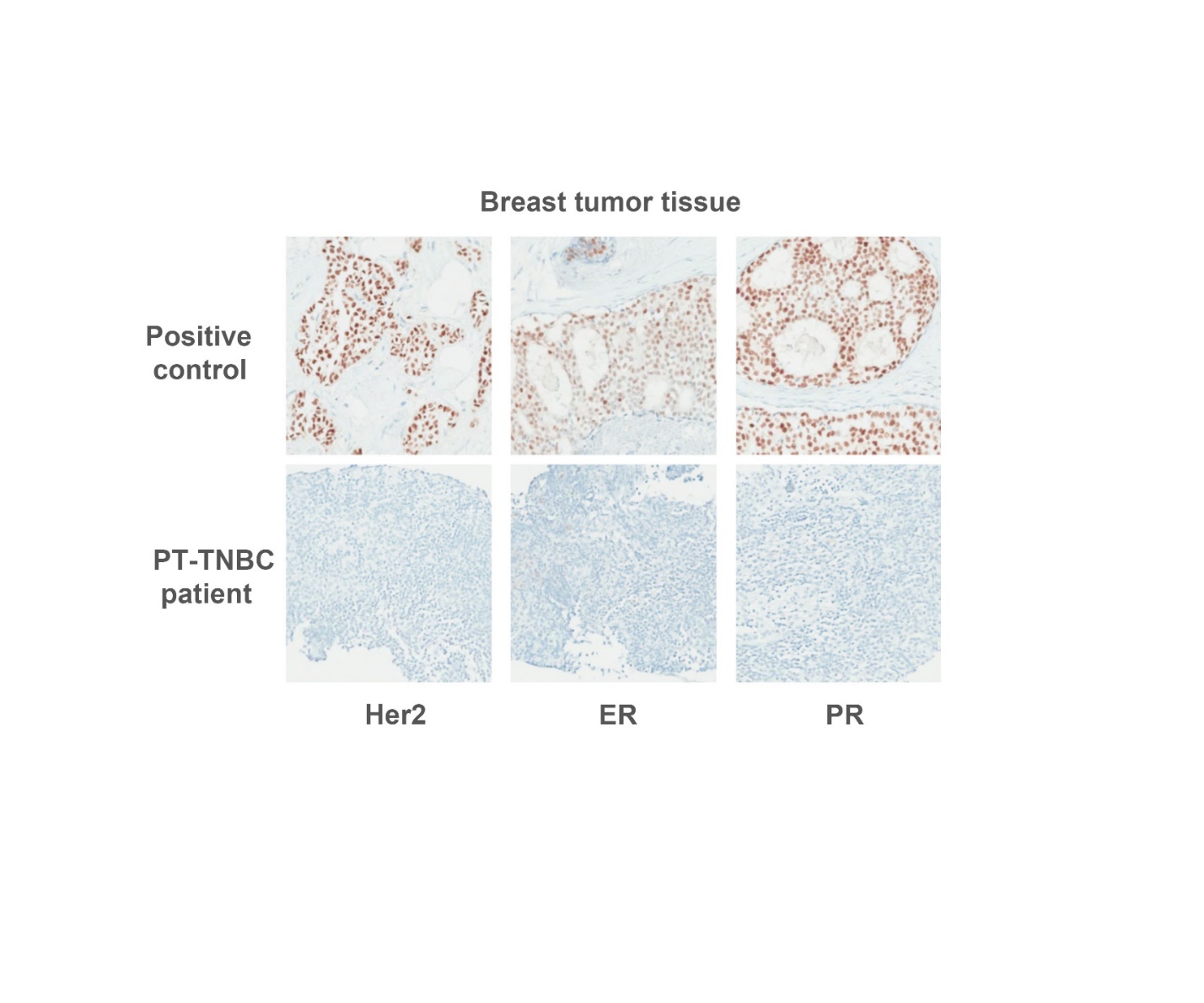


**Supplementary figure S2** : complete Western blot for PTHrP in MDA-MB-231 and PT-TNBC cells. KO: knock-out, Cont: control, MW: molecular weights. Arrow: PTHrP at 17 kDa.


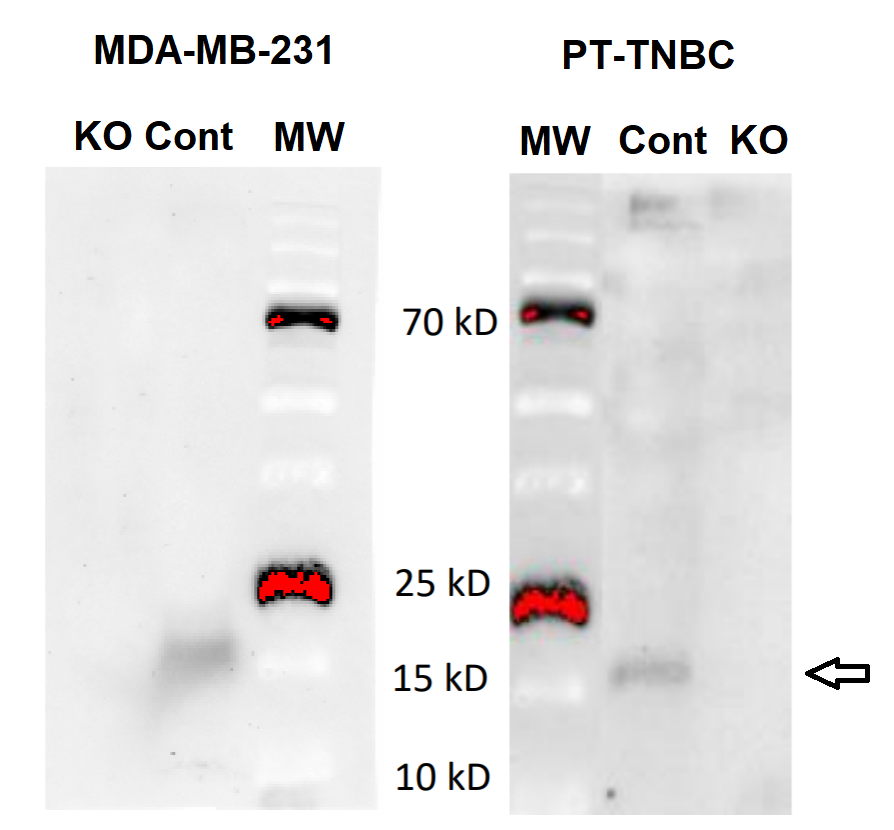


**Supplementary figure S3: Experimental protocol for *in vivo* experiments**. (A) Experimental protocol: Human MDA-MD-231 or PT-TNBC cells (*Pthlh* ^WT^) were injected (1 x 10^4^ cells in 15 ml of PBS) intra-tibially into 3- to 4-week-old BALB/C nu/nu female mice (n = 10 mice/group). Anti-PTHrP mAb injections (3 times/week) started at day 14 after intratibial TNBC cell injections and addressed established metastases. Total treatment time: 2 weeks (PT-TNBC) 3 weeks (MDA-MB-231). Animals were weighed once/week. Sacrifice was at day 35 for MDA-MB-TNBC-injected mice and day 28 for PT-TNBC-injected animals due to tumor size. (B,C) Time-wise weight gain in mAb-treated and untreated animals: mAb treatments did not affect the body weight of the mice. Dashes indicate confidence intervals. (n = 10, p < 0.5). Black circles: control; Green triangles: PTHrP mAb.
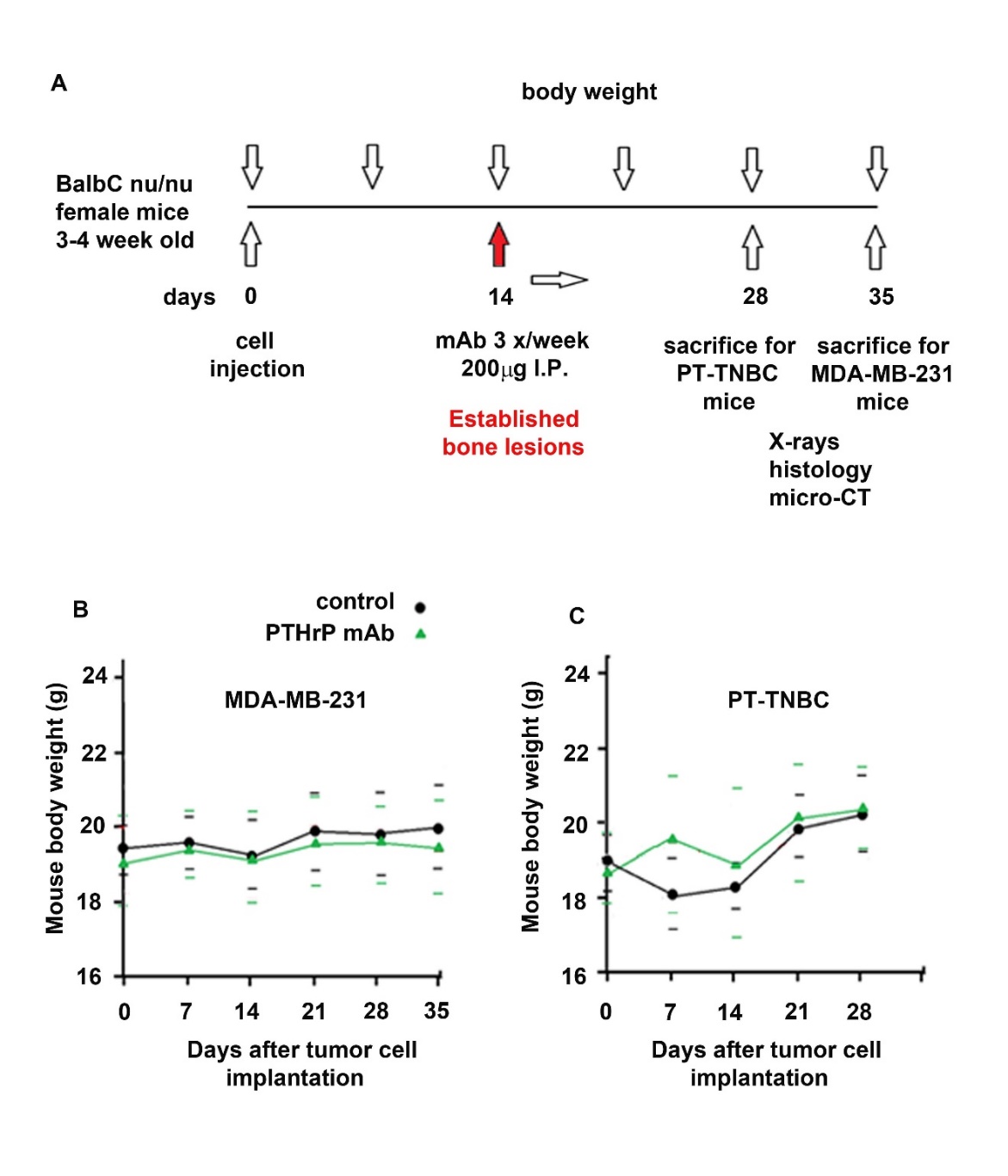


**Supplementary figure S4: Effect of anti-PTHrP mAb on normal bone.** Non-injected bone (A) (no cancer cells) is not statistically significantly affected by anti-PTHrP mAb treatment in terms of osteoblast (B), osteoclast numbers (C), or bone volume (D).


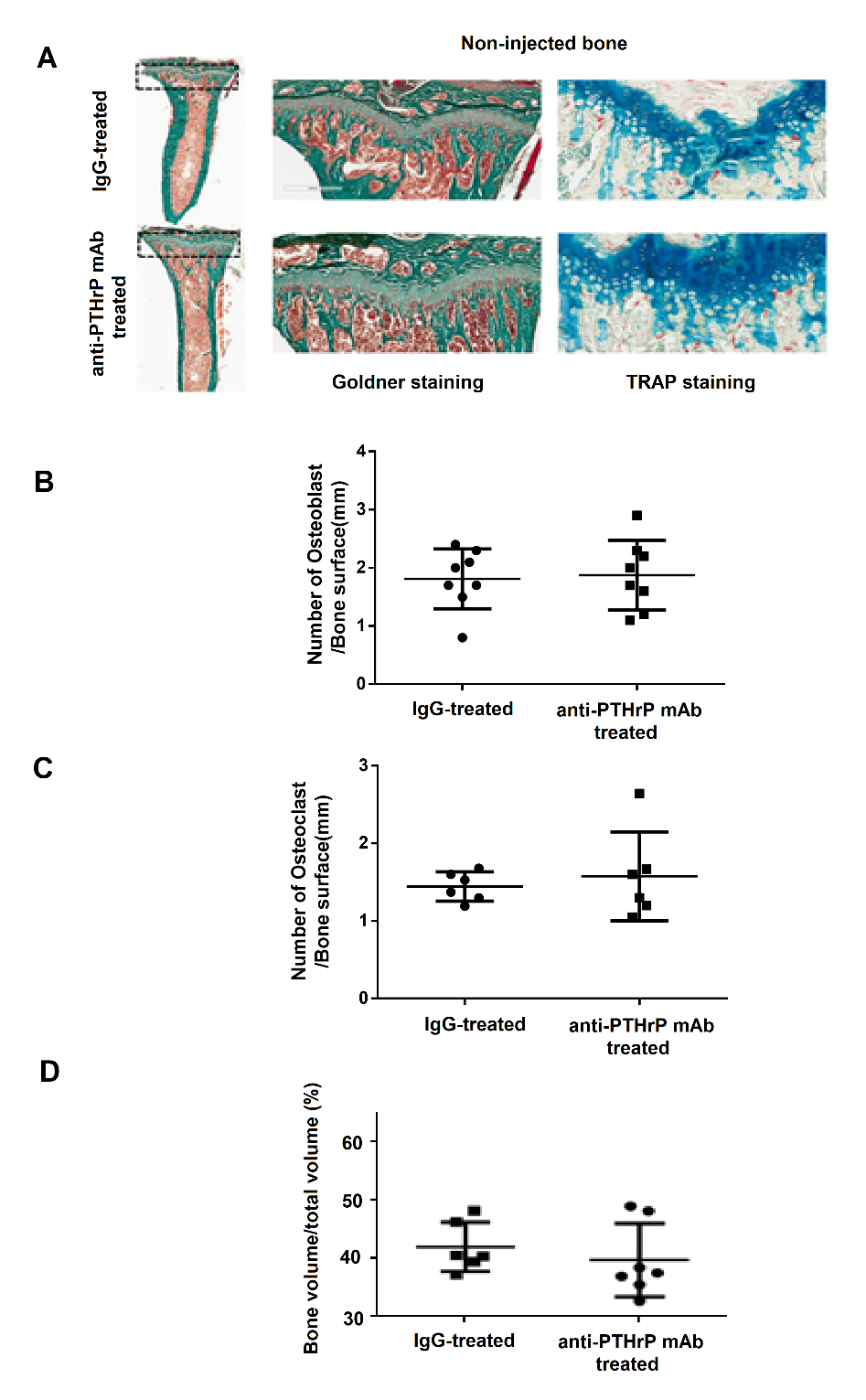

Supplement: Supplementary file 1 — Appendix S1. Supporting Information. Fig. S1 Fig. S2 Fig. S3 Fig. S4 Table S1 [file JBM4-6-e10587-s001.docx]
